# Trust Dynamics in Financial Decision Making: Behavioral Responses to AI and Human Expert Advice Following Structural Breaks

**DOI:** 10.3390/bs14100964

**Published:** 2024-10-17

**Authors:** Hyo Young Kim, Young Soo Park

**Affiliations:** 1 Department of Artificial Intelligence, Tech University of Korea, 237 Sangidaehak-ro, Siheung-si 15073, Republic of Korea; hyoyoung@tukorea.ac.kr; 2 College of Business Administration, Kookmin University, 77 Jeongneung-ro, Seongbuk-gu, Seoul 02707, Republic of Korea

**Keywords:** advice-taking behavior, trust erosion, human experts, artificial intelligence, financial shocks

## Abstract

This study explores the trust dynamics in financial forecasting by comparing how individuals perceive the credibility of AI and human experts during significant structural market changes. We specifically examine the impact of two types of structural breaks on trust: Additive Outliers, which represent a single yet significant anomaly, and Level Shifts, which indicate a sustained change in data patterns. Grounded in theoretical frameworks such as attribution theory, algorithm aversion, and the Technology Acceptance Model (TAM), this research investigates psychological responses to AI and human advice under uncertainty. This experiment involved 157 participants, recruited via Amazon Mechanical Turk (MTurk), who were asked to forecast stock prices under different structural break scenarios. Participants were randomly assigned to either the AI or human expert treatment group, and the experiment was conducted online. Through this controlled experiment, we find that, while initial trust levels in AI and human experts are comparable, the credibility of advice is more severely compromised following a structural break in the Level Shift condition, compared to the Additive Outlier condition. Moreover, the decline in trust is more pronounced for human experts than for AI. These findings highlight the psychological factors influencing decision making under uncertainty and offer insights into the behavioral responses to AI and human expert systems during structural market changes.

## 1. Introduction

Artificial Intelligence (AI) has become increasingly prevalent in financial forecasting, with decision makers relying more on AI-generated advice when formulating strategies and making decisions [1,2,3]. However, questions remain about the relative credibility of AI compared to traditional human expert advice, particularly during periods of significant market volatility. While previous research explored trust in AI and human experts under stable market conditions [4,5], the dynamics of trust during structural breaks in financial markets remain underexplored.

As global financial markets become more volatile and unpredictable, understanding how trust in AI and human experts shifts during market disruption has become crucial. Recent global crises, such as the COVID-19 pandemic and the ongoing conflict in Ukraine, have caused unprecedented disruptions in financial markets, highlighting the need to reassess the reliability of forecasting systems in such unpredictable environments [2,6]. The Russian–Ukrainian war, in particular, led to widespread financial instability, with significant impacts on commodities, stock markets, and foreign exchange rates. Stock market volatility spiked immediately following the invasion, with European and global markets showing sharp declines in synchronization with the conflict [7]. Commodity markets were especially affected, with wheat and nickel showing the most intense and sustained disruptions due to the strategic importance of Russia and Ukraine in the global supply chain. In addition, the war exacerbated global inflationary pressures that were already heightened by the COVID-19 recovery, particularly affecting energy and commodity prices, leading to supply chain disruptions and volatility in key markets [8]. These events emphasize the importance of investigating how structural breaks—defined as sudden and significant changes in market conditions—affect the perceived credibility of predictive advice [9].

In this study, we investigate trust dynamics in financial forecasting by comparing how individuals perceive the credibility of AI and human experts during significant structural changes. Specifically, we examine this comparison using two types of structural breaks: Additive Outliers and Level Shifts. An Additive Outlier represents a temporary anomaly, while a Level Shift indicates a more permanent shift in the underlying data pattern [10,11]. These different types of breaks are expected to have varying impacts on trust in AI versus human expert advice, potentially leading to different degrees of trust erosion.

The objectives of this research are threefold: (1) to compare trust levels in AI and human experts during normal market conditions; (2) to examine how different types of structural breaks—Additive Outlier and Level Shift—affect the credibility of AI and human experts; and (3) to identify which source of advice—AI or human experts—experiences a more pronounced decline in trust following a structural break and to explore the underlying mechanisms. Through a controlled experiment, we aim to provide empirical insights into these questions, thereby contributing to a deeper understanding of trust dynamics in financial forecasting during periods of market instability.

We structure the rest of the paper as follows: Section 2 reviews the relevant literature and formulates the hypotheses. Section 3 describes the experimental design and methodology, and Section 4 presents the analysis and results. Section 5 concludes with a discussion of our research.

## 2. Literature Review and Hypotheses Development

The emergence of Artificial Intelligence in complex predictive tasks, including financial forecasting, has sparked significant interest in understanding how AI compares to human experts in terms of trust. Prior research suggests that when AI and human experts are presented as reliable sources of advice, individuals tend to exhibit similar levels of trust in both, particularly in scenarios requiring intricate judgment. The Technology Acceptance Model (TAM) and Unified Theory of Acceptance and Use of Technology (UTAUT) provide strong theoretical foundations for understanding how individuals accept and trust technology. Venkatesh et al. [12] proposed that factors like performance expectancy, effort expectancy, and facilitating conditions heavily influence technology adoption and trust, offering a useful framework for understanding how individuals evaluate AI systems. These models suggest that as long as AI demonstrates competence and ease of use, it can garner levels of trust similar to human experts.

Logg et al. [4] found that people often appreciate algorithmic judgments as much as, or even more than, human judgments when the AI’s capabilities are effectively communicated. Furthermore, Dietvorst et al. [5] suggest that before any observed errors, the trust in AI can be comparable to that in human experts, indicating a baseline similarity in how advice from both sources is perceived. Jussupow et al. [13] also support this view, showing that, in fields like medical decision making, AI is trusted on par with human experts, particularly when it is perceived as enhancing the decision-making process. Similarly, Vodrahalli et al. [14] found that individuals use advice similarly for human and AI suggestions. Castelo et al. [15] further suggested that algorithm aversion is task-dependent: people are more likely to trust algorithms in tasks requiring objectivity but resist using them for subjective tasks, which may explain differences in trust across contexts. Finally, Dzindolet et al. [16] highlighted that initial trust is largely influenced by the perceived competence of both AI and human advisors, especially in predictive scenarios. Given this body of research, we hypothesize that participants will initially trust AI and human experts at similar levels in our study.

**Hypothesis 1.**  *There is no significant difference in initial trust levels between AI and human experts.*

A Level Shift represents a permanent change in the mean level of a time series, indicating a fundamental shift in the underlying data-generating process [10,11]. This sustained change poses a significant challenge for both AI and human experts, who must quickly adapt their predictions to align with the new reality. The resulting mismatch between expected and observed values can significantly reduce trust in the provided advice, as its reliability is questioned in the face of such a profound change [17]. If the forecasting model or expert fails to adjust to the new level, Level Shift can lead to prolonged inaccuracies, resulting in sustained errors [18]. These persistent errors can severely damage the credibility of the advice, leading to a more significant and lasting decline in trust. Longoni et al. [19] found similar results in the healthcare domain, where resistance to AI occurs due to its perceived inability to handle emotionally sensitive tasks, despite often outperforming human experts.

This dynamic is further explained by psychological theories such as attribution theory [20], which suggests that individuals attribute failures in judgment to internal or external causes. In the case of AI, its perceived objectivity might shield it from negative attributions, while human experts may be seen as responsible for their inability to adapt to new conditions. Additionally, automation bias [21]—the tendency to prefer human judgments over algorithms after observing errors—can exacerbate this trust erosion. The algorithm aversion concept, further studied by Dietvorst and Bharti [22], highlights that people often reject algorithms in uncertain domains due to their diminishing sensitivity to forecasting errors. Even if the AI consistently outperforms human experts, minor perceived mistakes can result in a disproportionate loss of trust.

In contrast, an Additive Outlier represents a temporary, isolated anomaly in the time series, not indicative of a permanent shift in the underlying process [23]. Participants are likely to perceive this anomaly as an exception rather than a systemic issue. Both AI and human experts are expected to resume accurate forecasting once the outlier passes. The error caused by an Additive Outlier is typically short-lived, and the time series returns to its original pattern after the outlier period ends, making it easier for both AI and human experts to regain or maintain trust [17]. Therefore, an Additive Outlier is less likely to cause a long-term loss of trust due to its temporary nature and the ability of both AI and human experts to quickly adjust.

**Hypothesis 2.**  *Following a structural break in the form of a Level Shift, trust in both AI and human experts will significantly decline. In contrast, trust in both AI and human experts will remain relatively stable following an Additive Outlier.*

Prior studies suggest that people often hold AI to a standard of perfection, leading them to be more forgiving of human errors while being more critical of AI mistakes [5]. Despite this higher standard, recent evidence also suggests that AI can maintain a more consistent level of perceived reliability under sustained disruptions because it is viewed as less prone to emotional or cognitive biases than human experts [13,24]. Further, AI systems are perceived as more consistent and less prone to biases, which helps maintain trust even in the face of disruptions, compared to human experts who might be affected by biases and slower adaptation processes [25]. Attribution theory explains this difference, as failures in AI are often attributed to the system’s limitations rather than the intent or competence of the designer, while human experts are judged more harshly for similar errors. This difference implies that after a structural break, such as a Level Shift, participants may perceive the failure of human experts to adjust to the new level as more problematic, resulting in a steeper decline in trust. Conversely, AI is often perceived as objective and neutral, relying solely on data rather than subjective judgment [25,26,27]. AI’s systematic, unbiased, and data-driven approach allows us to avoid a steep decline in trust. Therefore, we conjecture that the decrease in trust following a Level Shift will be more pronounced for human experts compared to AI.

**Hypothesis 3.**  *After a Level Shift, the decline in trust will be more pronounced for human experts compared to AI.*

**Figure 1:**
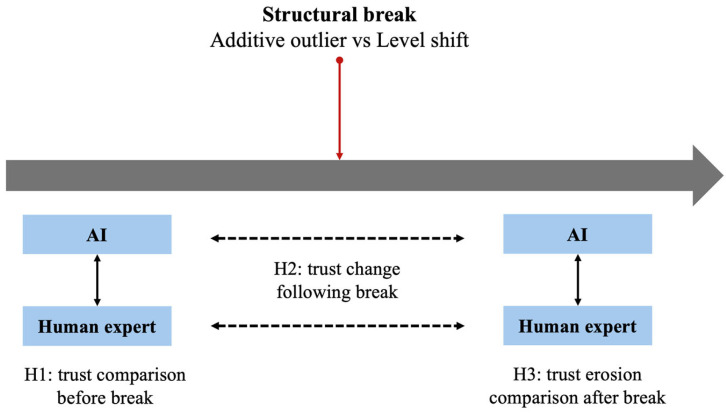
Research framework.

Figure 1 illustrates the conceptual research framework for this study.

## 3. Research Methods

### 3.1. Experimental Design

We used a 2 × 2 between-subject experimental design, varying the type of structural change (Additive Outlier vs. Level Shift) and the type of advisor (AI vs. human expert). The variations in structural change were referred to as conditions, while the variations in advisor type were referred to as treatments.

Participants were asked to make forecasts for stock prices over 30 independent rounds. In each round, they first made an “Initial Forecast” by reviewing historical stock price observations and then predicting the price one period ahead. Following this initial forecast, participants received advice from either an AI system or a human expert, depending on their assigned treatment. Importantly, both sources provided identical predictions but were framed as either an AI system or a human expert. Participants were then asked to adjust their initial forecasts based on the advice and submit a “Final Forecast.” This process was repeated for a total of 30 periods, specifically covering periods 61 to 90.

A structural break occurred in the 77th period of the time series. Before this break, stock prices were generated using the ARIMA(0,1,1) model, with both Additive Outlier and Level Shift conditions observing identical stock price realizations up to the break. The ARIMA (Autoregressive Integrated Moving Average) model was widely recognized in the previous literature as a reliable method for predicting future trends [28,29,30]. Specifically, the ARIMA(0,1,1) model was identified as one of the most effective models for forecasting stock prices [31,32]. This model represents a simple yet robust approach, where the time series is essentially a random walk with a lagged error term. The specific equation used in our study is as follows:

$$Y_{t}=Y_{t-1}+\varepsilon_{t}-{0.8\varepsilon }_{t-1},\;\varepsilon_{t}\sim N\left(0,1\right),$$

where the initial value $Y_{0}=15$, and $\varepsilon_{t}$ represents a white noise term.

As the structural break was introduced in the 77th period, the time series changed according to the specific condition assigned, either Level Shift or Additive Outlier. The specific models for these structural changes are defined as follows:

$$Level\;Shift:Z_{t}=\frac{\left(1-\theta B\right)}{\left(1-B\right)}\varepsilon_{t}+\frac{1}{\left(1-B\right)}∆I_{t}\left(m\right),$$

$$Additive\;Outlier:Z_{t}=\frac{\left(1-\theta B\right)}{\left(1-B\right)}\varepsilon_{t}+∆I_{t}\left(m\right),$$

where $\varepsilon_{t}\sim N\left(0,1\right),\;\theta =0.7,\;∆=10$, and the shift occurs at m=77.

In these models, B represents the backshift operator, and $∆I_{t}(m)$ denotes the intervention at the 77th period, which introduces either a sustained level shift or a one-time additive outlier into the time series. The initial condition for the series is $Z_{0}=10$.

We collected data from 157 participants (103 male and 54 female) using Amazon Mechanical Turk (MTurk), a platform that provides access to a global, geographically diverse pool of participants, enhancing the generalizability of our findings. This diversity ensures that this study’s results reflect broader perspectives on trust dynamics in financial forecasting across different cultural and economic backgrounds, rather than being limited to a specific country. Participants’ ages ranged from 20 to 63 years, with a mean age of 29.04 years. The experiment was programmed using the Shiny Apps platform, which is commonly used in laboratory experiments and behavioral analyses [33,34,35,36]. Before the experiment began, participants received detailed instructions regarding the information presented and their tasks. They were required to answer several comprehension questions to ensure they understood the experimental instructions before proceeding to the actual task. Additionally, participants completed a brief demographic questionnaire and a short survey before starting the experiment. The entire experiment took an average of 40 min to complete, with an average compensation of $3.25, which included a participation fee of $2. The screenshots of the software are in Appendix A.

### 3.2. Initial Trust Level

Before starting the experiment, we conducted a preliminary survey to evaluate participants’ baseline trust levels in both AI systems and human experts. This assessment was a critical component of our study, as it provided the foundation for understanding how trust changes in response to various experimental conditions. Participants were asked to rate their trust on a 7-point Likert scale, a widely recognized psychometric tool that allows for the nuanced measurement of attitudes and perceptions. On this scale, a rating of 1 indicated minimal trust, while a rating of 7 reflected maximum trust.

### 3.3. Weight of Advice

To evaluate how the source of advice—whether from an AI system or a human expert—affects the trust placed in that advice, and to examine how this trust changes following a structural break, we employed the “Weight of Advice” (WOA) metric. This measure, initially conceptualized by Harvey and Fischer [37], determines the extent to which individuals adjust their forecasts in response to the advice received. In essence, WOA serves as an indicator of how much the advice is trusted and subsequently relied upon. The WOA is calculated using the following:

$$WOA=\frac{Final\;Forecast-Initial\;Forecast}{Advice-Initial\;Forecast}$$

This WOA ratio yields a value ranging from 0 to 1, representing the extent to which the final decision reflects the input provided by the advisor. A WOA value of 0 signifies that the decision maker has disregarded the advice entirely, adhering strictly to their initial forecast. Conversely, a WOA of 1 indicates complete alignment with the advice, signifying full adoption. A value of 0.5 suggests that the individual has balanced their final judgment equally between their initial forecast and the advice, positioning the final forecast halfway between the two. Therefore, when WOA approaches 1, it signals that the decision maker heavily relied on the advice, indicating a high degree of trust in the source. On the other hand, a WOA closer to 0 suggests that the decision maker favored their initial judgment, reflecting a lower level of trust in the advice.

In cases where the final forecast deviated beyond the bounds established by the initial forecast and the advice, we applied winsorization to the WOA values [38,39,40]. In this approach, any WOA value calculated to be less than 0 was adjusted to 0, and any value greater than 1 was adjusted to 1. Winsorization ensures that the WOA metric remains both meaningful and interpretable, preventing outliers from distorting the analysis and maintaining the integrity of the measurement of advice-taking behavior. Table 1 provides a summary of statistics and empirical distribution of the average WOA for each participant in our study.

### 3.4. Model Specifications

To examine whether the changes in the WOA following a structural break are moderated by the type of advice received—whether from an AI system or a human expert—we employed a Difference-in-Difference (DID) model. This methodology provides a rigorous framework for assessing the differential impact of structural changes on trust by comparing shifts in the WOA across AI and human expert formats. The empirical model we used is as follows:

(1) $${WOA}_{it}=\alpha_{i}+\beta_{1}\left({Human\;expert}_{i}\times {PostBreak}_{t}\right)+Controls+\epsilon_{it}$$

${WOA}_{it}$ denotes the WOA for individual i at time t. The term $\alpha_{i}$ captures individual-specific fixed effects, which account for time-invariant unobserved heterogeneity among participants. By controlling these fixed effects, the model adjusts for personal attributes that remain constant over time but could influence an individual’s baseline level of trust in advice. This includes demographic factors such as gender, age, or other inherent participant characteristics.

The interaction term ${Human\;expert}_{i}\times {PostBreak}_{t}$ is pivotal to our analysis, as it identifies the differential impact of the structural break on WOA based on the source of advice. The variable ${Human\;expert}_{i}$ is a binary indicator, coded as 1 if the advice was provided by a human expert and 0 if it was generated by an AI system. Similarly, the ${PostBreak}_{t}$ variable is a binary indicator that takes the value of 1 for observations recorded after the structural break and 0 for those before the break.

The coefficient $\beta_{1}$ serves as our DID estimator and is crucial for quantifying the differential change in WOA attributed to the structural break. Specifically, $\beta_{1}$ captures how the structural break influenced trust in advice differently between AI and human expert sources. A statistically significant coefficient would indicate that the structural break had a distinct impact on how much individuals relied on advice, depending on whether the advice was AI-driven or provided by a human expert. The error term $\epsilon_{it}$ captures the unexplained variation in WOA, accounting for random fluctuations in how participants weigh advice.

In addition, we included control variables to account for factors that could influence WOA. First, we controlled for the performance of the advice in the previous period, as poor performance might lead to reduced reliance on subsequent advice. This was incorporated into the regression model using the Absolute Percentage Error (APE) from the previous period’s advice (LagAPE). We also controlled for the extent to which participants relied on advice in the previous period, represented by the previous period’s WOA (Lag$WOA_{i,t-1}$), to capture any persistence in their advice-taking behavior.

## 4. Analysis Results and Discussions

### 4.1. Preliminary Analysis: Initial Forecasting Performance

As the first step in our analysis, we conducted preliminary tests to assess participants’ initial forecasting performance. This step was crucial to ensure that any differences observed later in the experiment could be attributed to the experimental conditions rather than inherent differences in the participants’ predictive abilities.

To this end, we compared participants’ initial forecasting performance across the different experimental treatments. Forecasting performance was measured using the Absolute Percentage Error (APE), calculated as the percentage difference between the actual value and the forecasted value. Table 2 shows the APE for each treatment, along with the contrasts between AI system and human expert treatments.

In both the Level Shift and Additive Outlier conditions, the initial forecasting performance did not show significant differences between the AI and human expert treatments. In the Level Shift condition, the APE for the human expert treatment was 15.307 percentage points lower than that for the AI treatment; however, this difference was not statistically significant (p=0.209). Similarly, in the Additive Outlier condition, the APE for the human expert treatment was 7.548 percentage points higher than that for the AI treatment, but again, this difference was not statistically significant (p=0.648).

By establishing that there were no significant differences in APE during the initial period, we confirmed that the experiment was well balanced. This validation of the experimental design provides a solid foundation for the subsequent analysis, ensuring that any observed effects can be attributed to the experimental treatments rather than pre-existing differences in forecasting abilities.

### 4.2. Comparison of Initial Trust Level

To test Hypothesis 1, which posits that there is no significant difference in initial trust levels between AI and human experts, we first examined whether any such differences existed at the outset of the experiment across the two treatment conditions: AI and human expert. Table 3 presents the initial trust levels for each treatment, along with the contrasts between these treatments.

Our analysis of the initial trust levels revealed no statistically significant differences between the AI and human expert treatments in either the Level Shift or Additive Outlier conditions. Although the average trust level was marginally higher for the human Expert treatment in both conditions, the contrasts between the two treatments were not statistically significant. Specifically, in the Level Shift condition, the trust level contrast was 0.244, and in the Additive Outlier condition, it was 0.014. Neither of these contrasts reached a level of statistical significance (p=0.605, p=0.973), suggesting that participants commenced the experiment with comparable levels of trust in both AI and human experts.

These findings support our Hypothesis 1, confirming that there were no significant pre-existing differences in trust between the two sources of advice at the start of the experiment. This result aligns with the prior research indicating that, when presented as competent and reliable, AI and human experts are trusted at similar levels [5,14]. Our study extends these findings by demonstrating that this equivalence in trust also holds within the context of financial forecasting. Furthermore, this baseline similarity is crucial for ensuring that any subsequent changes in trust observed during the experiment can be attributed to the experimental manipulations rather than to any inherent biases or predispositions toward AI or human experts.

### 4.3. Changes in Trust Following the Structural Break

Our second hypothesis posits that the decrease in trust following a structural break will be more significant in the case of a permanent change (Level Shift) compared to a temporary change (Additive Outlier). We further hypothesized that this effect would be consistent across both AI and human expert treatments.

To test this hypothesis, we analyzed changes in trust levels, as measured by the WOA, before and after the structural break within each treatment condition. Specifically, we divided our sample into two sub-samples based on the timing of the structural break, which occurred in the 77th period. We then examined whether the WOA significantly differed between the periods before and after the break. The results are presented in Table 4.

Our analysis shows a significant decrease in WOA following the structural break under the Level Shift condition for both AI and human expert treatments. Specifically, in the AI treatment, WOA decreased by 0.065 (p=0.06), while in the human expert treatment, the decrease was more pronounced, with a reduction of 0.151 (p ≤0.01). These results suggest that participants’ trust in advice, whether from AI or human experts, significantly diminishes when faced with a sustained change in the predictive environment.

In contrast, under the Additive Outlier condition—where the structural break represents a temporary anomaly rather than a lasting shift—no significant changes in WOA were observed for either treatment. This lack of significant change indicates that participants maintained their trust in both AI and human experts despite the short-term disruption, perceiving these anomalies as isolated incidents rather than as indicators of broader reliability issues.

Overall, these findings support our second hypothesis by highlighting the nuanced ways in which different types of predictive errors—persistent versus temporary—affect trust in advice. The significant reduction in WOA under the Level Shift condition underscores that enduring shifts in predictive accuracy can diminish the perceived reliability of advice, leading to a notable decline in trust. In contrast, the stability of WOA in response to Additive Outliers suggests that individuals are more forgiving of occasional errors, maintaining their trust in advice when the disruption is perceived as an outlier rather than a systemic issue. These insights provide a deeper understanding of how the nature of predictive errors influences the dynamics of advice-taking behavior.

### 4.4. Impact of Structural Break on Trust: AI System vs. Human Expert

In our previous analysis, we found a significant reduction in trust under the Level Shift condition for both AI and human expert treatments following the structural break. We now seek to determine which treatment—AI or human expert—experienced a more pronounced decrease in trust after the structural break. Hypothesis 3 posits that following a structural break in the Level Shift condition, the decrease in trust will be more salient for human expert compared to AI systems. To test this hypothesis, we employed the Difference-in-Difference (DID) method. The results of the DID regression analysis are presented in Table 5.

In the Level Shift condition (Columns 1 and 2), the coefficient of interaction term ${Human\;expert}_{i}\times {PostBreak}_{t}$ is negative and statistically significant, with coefficients of −0.151 (p ≤0.01) and −0.111 (p ≤0.01), respectively. These results indicate that following the structural break, there was a substantial decrease in WOA for the human expert treatment compared to AI treatment. This result suggests that participants significantly reduced their reliance on advice after experiencing a persistent change, especially when the advice was provided by a human expert rather than an AI system. The inclusion of control variables does not change the significance and direction of this relationship, underscoring the robustness of the findings (Column 2).

In contrast, under the Additive Outlier condition, the interaction term is positive but not statistically significant in either model (Columns 3 and 4), with coefficients of 0.034 (p=0.256) and 0.044 (p=0.109), respectively. This result indicates that the temporary nature of the structural break did not lead to a significant change in trust in human expert advice relative to AI. The result also suggests that participants were more resilient to transient anomalies, maintaining consistent levels of trust in both AI and human expert advice despite the brief disruption.

In summary, these findings support Hypothesis 3, showing that following a structural break in the Level Shift condition, trust in human experts decreased more significantly than trust in AI systems. This finding suggests that human advisors may be more vulnerable to trust erosion in situations where predictive accuracy is persistently challenged.

## 5. Conclusions

We investigate the dynamics of trust in financial forecasting, particularly how individuals perceive the credibility of AI versus human experts in the face of significant structural changes. Through a controlled experimental design, we examined the impact of two types of structural breaks—Additive Outliers and Level Shifts—on trust levels in AI and human expert advice.

Our findings reveal that while initial trust in AI and human experts is comparable, the occurrence of a Level Shift leads to a more substantial decline in trust, particularly in human experts. This outcome suggests that participants may perceive human experts as less reliable when predictive accuracy is persistently challenged by fundamental changes in the data. Conversely, the resilience of trust in AI systems, even after such disruptions, highlights their perceived objectivity and consistency, which may shield them from the same level of trust erosion faced by human experts. In contrast, the impact of Additive Outliers on trust was minimal, with no significant difference observed between AI and human experts. This lack of significant difference indicates that participants were more forgiving of temporary anomalies, maintaining stable trust levels regardless of the source of advice. These results underscore the nuanced ways in which different types of structural breaks can influence the dynamics of trust in AI versus human experts.

### 5.1. Theoretical Contributions

This research contributes to the literature on trust dynamics in decision making by examining how individuals evaluate the credibility of AI versus human expert advice under conditions of structural disruption. Previous studies primarily focused on trust in AI and human experts in stable environments [25,41,42], but this study expands the understanding of how trust shifts during significant market disturbances, specifically Additive Outliers and Level Shifts. The finding that trust in human experts erodes more severely after a Level Shift highlights a critical aspect of decision-making psychology, where individuals may attribute sustained prediction failures to human fallibility rather than systematic limitations. Conversely, the relative resilience of trust in AI following these breaks underscores the perceived objectivity and adaptability of AI, even when held to higher performance standards. These insights advance theoretical models of trust by incorporating the role of structural breaks and differentiating between transient versus permanent market anomalies, offering a more comprehensive understanding of trust behavior in dynamic environments.

### 5.2. Practical Implications

This study has important practical implications for organizations and managers who rely on automated systems for decision making, particularly in the context of financial forecasting. Our findings suggest that the systematic and data-driven nature of AI enables it to adapt more effectively to sustained disruptions, thus preserving trust more efficiently than human experts. These insights offer valuable guidance for the design and implementation of AI systems in financial forecasting and beyond. Organizations should consider designing forecasting systems that leverage the strengths of AI—its objectivity, adaptability, and resilience to disruptions—while also providing transparency to enhance trust during periods of volatility.

As AI continues to play an increasingly prominent role in decision-making processes, understanding how trust in these systems is maintained or eroded during times of instability is crucial for their successful integration. Ensuring that AI systems are transparent in their operations and capable of adapting to significant changes in data patterns may help sustain user trust, especially in volatile markets.

### 5.3. Limitations and Opportunities for Future Research

Despite its novel contributions, this study has several limitations that open up several interesting avenues for future research. First, the scope of this research is limited to specific types of structural breaks, namely Additive Outliers and Level Shifts, and focuses solely on the immediate trust response to these disruptions. However, individual heterogeneity—such as gender, education level, work experience, and risk preferences (e.g., risk aversion)—could play a significant role in shaping trust dynamics in AI versus human experts. Investigating how these individual differences affect trust responses would provide deeper insights into the nuanced psychological factors influencing decision making.

Second, while this study only focused on the stock price forecasting scenario, there is significant potential to expand the scope to other forecasting domains. Areas such as economic trends, climate change predictions, or healthcare diagnostics present opportunities for examining whether similar trust dynamics hold in diverse and highly impactful contexts. Broadening the application of this research would not only enhance the generalizability of the findings but also provide practical relevance for decision makers across various industries.

Third, conducting experiments in real-world settings would offer deeper insights and lead to more actionable conclusions. In real-world decision making, a multitude of additional factors and complexities come into play that were beyond the scope of this study [43,44]. For example, financial forecasting in practice often deals with heightened uncertainty, relies on probabilistic estimates, and incorporates richer, more detailed contextual information [9,45,46,47]. These real-world conditions could have a significant impact on how trust in AI versus human experts is formed, maintained, and potentially eroded. By integrating these practical complexities, future research could better reflect the intricate nature of trust dynamics, thus providing more precise and relevant conclusions for real-world applications.

Finally, future studies could also investigate how AI transparency and explainability impact the maintenance and recovery of trust following structural breaks. Recent research showed that providing clear explanations for errors can enhance trust in decision-making systems [48,49]. As suggested by Arrieta et al. [48], explainable artificial intelligence (XAI) plays a crucial role in fostering trust by offering transparency and clarity on how AI systems make decisions. Studies could explore whether providing more detailed information about AI’s decision-making processes or offering explanations for predictions could mitigate the erosion of trust during periods of market instability. For example, transparent AI models that explain their reasoning could help users understand why certain predictions are made, especially during periods of volatility such as Level Shifts. This enhanced understanding may enable users to feel more confident in AI-driven advice and potentially reduce their reliance on human experts when trust in AI is maintained through clear and interpretable explanations. By examining how explainability influences trust in AI under dynamic market conditions, future research can provide valuable insights into designing AI systems that are both effective and trusted by users in high-stake environments like financial forecasting. By integrating explainable AI, users could gain a better understanding of the decision-making processes behind AI-generated advice, potentially reducing trust erosion and promoting faster trust recovery after disruptions.

## Figures and Tables

**Table 1:** Summary statistics: weight of advice.

Condition	Treatment	Average Weight of Advice				
		Mean	SD	25%	50%	75%
Level Shift	AI	0.432	0.279	0.227	0.386	0.636
	human expert	0.405	0.265	0.180	0.407	0.609
Additive Outlier	AI	0.470	0.344	0.121	0.501	0.756
	human expert	0.456	0.296	0.220	0.462	0.591

**Table 2:** Initial forecasting performance.

Condition	Treatment	N	Initial Forecast Performance	
			APE	Contrast ^a^
Level Shift	AI	45	36.794	
	human expert	36	21.487	−15.307
Additive Outlier	AI	36	33.765	
	human expert	40	41.314	7.548

Note: ^a^ Contrasts reflect the difference between an outcome in AI and human expert treatments. The null hypothesis is that contrast = 0. Null hypothesis was tested using a *t*-test, * p ≤0.1; ** p ≤0.05; *** p ≤0.01.

**Table 3:** Initial Trust Level.

Condition	Treatment	N	Initial Trust Level	
			Trust	Contrast ^a^
Level Shift	AI	45	2.644	
	human expert	36	2.889	0.244
Additive Outlier	AI	36	2.361	
	human expert	40	2.375	0.014

Note: ^a^ Contrasts reflect the difference between an outcome in AI and human expert treatments. The null hypothesis is that contrast = 0. Null hypothesis was tested using a *t*-test, * p ≤ 0.1; ** p ≤ 0.05; *** p ≤ 0.01.

**Table 4:** Changes in WOA after the structural break.

Condition	Treatment	WOA		
		Before Break	After Break	Difference
Level Shift	AI	0.463	0.397	0.065 *
	human expert	0.475	0.324	0.151 ***
Additive Outlier	AI	0.474	0.466	0.008
	human expert	0.440	0.474	−0.034

Note: The null hypothesis is that difference in WOA before and after the break is ≤0. Null hypothesis was tested using a *t*-test, * p ≤ 0.1; ** p ≤ 0.05; *** p ≤ 0.01.

**Table 5:** Difference in Difference (DID) regression results.

Variables	Level Shift Condition		Additive Outlier Condition	
	(1)	(2)	(3)	(4)
DID (${Human\;expert}_{i}\times {PostBreak}_{t}$)	−0.151 ***	−0.111 ***	0.034	0.044
	(−2.90)	(−2.74)	(1.15)	(1.62)
Lag APE		−0.000		−0.000
		(−0.06)		(−1.02)
Lag WOA		0.231 ***		0.052
		(6.04)		(1.58)
Observations	2430	2349	2280	2204
Individuals	81	81	76	76
R-squared	0.012	0.255	0.002	0.187

Notes: Standard errors were robust to heteroscedasticity and clustered at the individual level. *t*-statistics are in parentheses. Significance at the 10%, 5%, and 1% levels is indicated by *, **, and ***, respectively. Constants are not reported.

**Figure A1:**
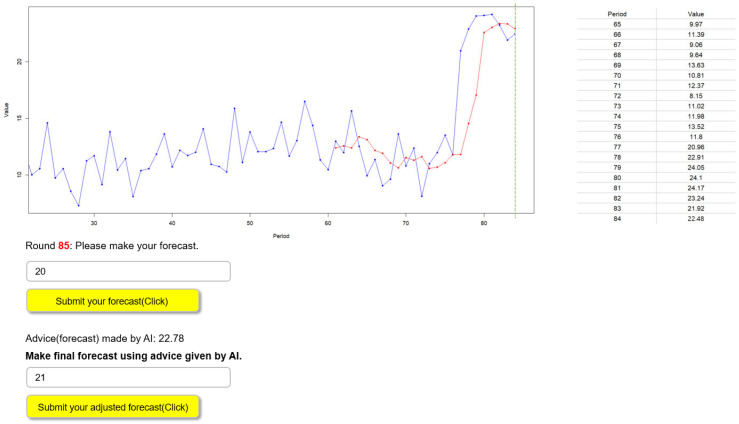
Screenshots of software—level shift condition and AI treatment. The blue line shows the actual stock price and the red line shows the advice (forecast).

## Data Availability

The data supporting the findings of this study can be obtained from the corresponding author upon request.
